# Variceal bleeding is aggravated by portal venous invasion of hepatocellular carcinoma: a matched nested case-control study

**DOI:** 10.1186/s12885-020-07708-1

**Published:** 2021-01-05

**Authors:** Jihye Lim, Ha Il Kim, Eunju Kim, Jiyoon Kim, Jihyun An, Seheon Chang, Seon-Ok Kim, Han chu Lee, Yung Sang Lee, Ju Hyun Shim

**Affiliations:** 1 Department of Gastroenterology, Asan Medical Center, University of Ulsan College of Medicine, 88, Olympic-ro 43-gil, Songpa-gu, Seoul, 05505 South Korea; 2 Gastroenterology, Kyung Hee University Hospital, Seoul, Republic of Korea; 3 Gastroenterology, Department of Internal Medicine, Haeundae Paik Hospital, College of Medicine, Inje University, Busan, Republic of Korea; 4 Gastroenterology, Hanyang University College of Medicine, Guri, Gyeonggi-do Republic of Korea; 5 Internal Medicine, Myongji St. Mary’s Hospital, Seoul, Republic of Korea; 6 Biostatistics and Clinical Epidemiology, Asan Medical Center, University of Ulsan College of Medicine, Seoul, Republic of Korea; 7 Asan Liver Center, Asan Medical Center, University of Ulsan College of Medicine, Seoul, Republic of Korea

**Keywords:** Hepatocellular carcinoma, Portal vein thrombosis, Esophageal varices, Variceal bleeding, Risk factor

## Abstract

**Background:**

We hypothesized that portal vein tumor thrombosis (PVTT) in hepatocellular carcinoma (HCC) increases portal pressure and causes esophageal varices and variceal bleedings. We examined the incidence of high-risk varices and variceal bleeding and determined the indications for variceal screening and prophylaxis.

**Methods:**

This study included 1709 asymptomatic patients without any prior history of variceal hemorrhage or endoscopic prophylaxis who underwent upper endoscopy within 30 days before or after initial anti-HCC treatment. Of these patients, 206 had PVTT, and after 1:2 individual matching, 161 of them were matched with 309 patients without PVTT. High-risk varices were defined as large/medium varices or small varices with red-color signs and variceal bleeding. Bleeding rates from the varices were compared between matched pairs. Risk factors for variceal bleeding in the entire set of patients with PVTT were also explored.

**Results:**

In the matched-pair analysis, the proportion of high-risk varices at screening (23.0% vs. 13.3%; *P* = 0.003) and the cumulative rate of variceal bleeding (4.5% vs. 0.4% at 1 year; *P* = 0.009) were significantly greater in the PVTT group. Prolonged prothrombin time, lower platelet count, presence of extrahepatic metastasis, and Vp4 PVTT were independent risk factors related to high-risk varices in the total set of 206 patients with PVTT (Adjusted odds ratios [95% CIs], 1.662 [1.151–2.401]; 0.985 [0.978–0.993]; 4.240 [1.783–10.084]; and 3.345 [1.457–7.680], respectively; Ps < 0.05). During a median follow-up of 43.2 months, 10 patients with PVTT experienced variceal bleeding episodes, 9 of whom (90%) had high-risk varices. Presence of high-risk varices and sorafenib use for HCC treatment were significant predictors of variceal bleeding in the complete set of patients with PVTT (Adjusted hazard ratios [95% CIs], 26.432 [3.230–216.289]; and 5.676 [1.273–25.300], respectively; Ps < 0.05).

**Conclusions:**

PVTT in HCC appears to increase the likelihood of high-risk varices and variceal bleeding. In HCC patients with PVTT, endoscopic prevention could be considered, at least in high-risk variceal carriers taking sorafenib.

## Highlights

Hepatocellular carcinoma with portal vein tumor thrombosis may increase the likelihood of developing high-risk varices and variceal bleeding.

The presence of high-risk varices and sorafenib use for hepatocellular carcinoma treatment were significant predictors of variceal bleeding.

## Background

Variceal hemorrhage is one of the main causes of non-cancer-related deaths in cirrhotic patients with hepatocellular carcinoma (HCC) [1–3]. Portal hypertension-driven excessive wall tension is a substantial contributor to esophago-gastric variceal rupture [4, 5].

Portal vein tumor thrombosis (PVTT) has a significant effect on the prognosis of HCC patients, resulting in a short survival time comparable or often inferior to metastatic patients. Accordingly, these two HCC categories are both classified as advanced stage (stage C) based on the Barcelona Clinic Liver Cancer (BCLC) system [6, 7]. Despite a lack of data on direct measurement of portal pressure in patients with HCC invading the portal vasculature, it is plausible that PVTT would secondarily enhance resistance and pressure in portal veins. Moreover, PVTT is robustly associated with variceal bleeding in HCC patients [8, 9]. as it is in cirrhotic patients with benign portal vein thrombosis [10]. With the exception of individuals with mild liver stiffness and normal platelet count who have a very low probability of high-risk varices, routine screening endoscopy for examining esophago-gastric varices is formally recommended for almost all cirrhotic patients. Treatment with non-selective beta-blockers (NSBBs), or endoscopic variceal ligation (EVL), are recommended for non-bleeders with varices, depending on the severity of the varices [5, 11, 12]. However, there is no consensus about variceal evaluation and prevention specifically targeting HCC patients, who generally have a greater risk of developing varices and associated hemorrhagic events, and there are no current guidelines [13].

In view of the relevant practical and strategic needs, we investigated the incidence of subclinical varices on endoscopy, especially in the esophagus, in the initial work-ups of a set of cases with HCC and PVTT. We also compared bleeding rates from the varices or overall upper gastrointestinal tract during the HCC treatment or follow-up periods in a matched control set without PVTT. Potential risk factors for variceal bleeding in patients with HCC accompanied by PVTT were also explored.

## Methods

### Study population

**Fig. 1:**
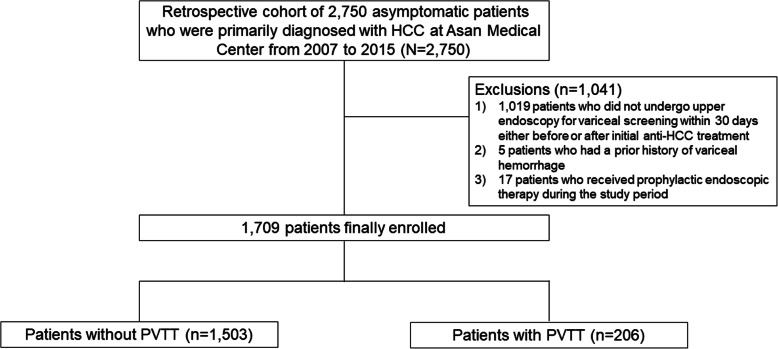
Flowcharts for patient selection

This retrospective study included a total of 2750 patients originally diagnosed as having HCC without any cancer-related symptoms at the Asan Medical Center, South Korea, between January 2007 and December 2015. The diagnosis of HCC was based on typical contrast-enhanced imaging criteria and/or pathological proof according to global practice guidelines [14–16]. Among these silent HCC patients, we excluded the following: 1) 1019 patients who did not undergo upper endoscopy for variceal screening within 30 days before or after initial anti-HCC treatment; 2) five who had a prior history of variceal hemorrhage; and 3) 17 who received prophylactic endoscopic therapy during the study period. The 1709 patients who were finally enrolled comprising 206 patients with PVTT and 1503 without PVTT (Fig. 1).

### Definitions of major parameters

High-risk varices were defined as large/medium varices (> 5 mm) or small varices (≤5 mm) with red-color signs, such as cherry-red spots, hematocystic spots, and red wale markings in the esophagus, which are known to be associated with a high likelihood of variceal hemorrhage [5, 17]. In addition, degree of PVTT was classified based on location and extension by contrast-enhanced computed tomography or magnetic resonance imaging as established by the classification system of the Liver Cancer Study Group of Japan [6]: 1) Type Vp1 was defined as tumor thrombus formation by microscopy; 2) type Vp2 was defined as tumor thrombus involving the segmental branch of the portal vein or above; 3) type Vp3 was defined as tumor thrombus involving large branches of the portal vein; and 4) type Vp4 was defined as tumor thrombus involving the main trunk of the portal vein.

### Definitions of study endpoints

Variceal bleeding was defined as a hemorrhagic event from the esophageal tract, with or without gastric varices identified by endoscopy, or with presence of large esophageal varices with blood in the stomach and no other visible bleeding foci. Overall bleeding was defined as any hemorrhagic episode arising from the upper gastrointestinal tract, including esophagus, stomach, and duodenum. Overall survival was calculated from the date of initiation of HCC treatment to death from any cause, or the date of last follow-up.

### Clinicopathological variables

The following parameters were examined as clinical and pathological variables at initial HCC diagnosis that predict the presence of high-risk varices or bleeders: 1) patient-related factors including age, gender, hypertension, etiology of underling liver disease, Child-Pugh class, laboratory test data, ascites, and model for end-stage liver disease (MELD) score; 2) tumor-related factors including number of tumors, maximal size of tumors, serum alpha-fetoprotein (AFP) level, presence of extrahepatic metastasis, and extent of PVTT; and 3) treatment-related factors including NSBBs (specifically propranolol and carvedilol) and anti-HCC therapeutic methods (transarterial chemoembolization [TACE], radiotherapy, and sorafenib). All relevant information was obtained through our hospital’s integrated healthcare system, consisting of a Picture Archiving Communication System and an Electronic Medical Record system (i.e., the Asan Medical Information System: AMIS). This complied with our Hospital Evaluation Program and Health Insurance Portability and Accountability Act (HIPAA) Standard Operating Procedures [18].

### Statistical analysis

Statistical analysis was performed with SAS (version 9.4, SAS Institute, Inc., Cary, NC, USA) and R (version 3.6.0, https://www.r-project.org) software. The chi-square test or Fisher’s exact test was used for categorical variables, and the t-test or Mann-Whitney test for continuous variables. Individual matching (1,2) by Greedy algorithm for age (± 5 years), gender, hypertension, hepatitis B virus (HBV) infection, prothrombin time (PT), platelet, aspartate aminotransferase (AST), alanine aminotransferase (ALT), creatinine, ascites, and Child-Pugh class was performed to balance selection differences and reduce selection bias. Tumor burden and therapeutic method for HCC, which depended directly on the presence or absence of PVTT were excluded from the matching variables. Using potential covariates that could affect clinical characteristics, we generated pairs of patients with and without PVTT. To compare the prevalence of varices in the matched set, logistic regression was performed with Generalized Estimating Equations (GEE), and the risks of variceal hemorrhage were compared with a Cox regression model, with robust standard errors, that accounted for the clustering of matched pairs. Subgroup analyses were performed for all HCC patients with PVTT to examine factors related to the formation of high-risk varices and variceal bleeding. Univariate and multivariable logistic regression analyses were performed to identify independent risk factors for high-risk varices. When evaluating the characteristics associated with variceal hemorrhage, a Cox proportional hazards model was used to establish the hazard ratio (HR) with a confidence interval (CI) of 95%. The Cox model for overall survival included variceal hemorrhage as the time-dependent covariate. Multivariable analyses were performed by backward elimination using variables with *P*-values of < 0.20 in the univariate analysis. *P*-values < 0.05 were considered statistically significant.

## Results

### Pre-endoscopic characteristics of the pooled and matched cohorts

The main demographic and clinical data of the pooled cohort are shown in Table 1. The mean age of the 1709 enrolled patients was 57.0 ± 9.3 years, and 1392 (81.5%) were men. Liver cirrhosis was observed in all the patients. Most patients had HBV infection (84.1%) and Child-Pugh class A liver disease (91.5%). The mean MELD score was 8.2 ± 2.3. There were 451 (26.4%) hypertensive patients. The PVTT group was younger (means ± standard deviation [SD], 55.5 ± 9.5 vs. 57.2 ± 9.2, *P* = 0.013), and included more men (87.9% vs. 80.6%, *P* = 0.012) and infiltrative-type tumors (1.5% vs. 36.9%, *P* < 0.001). Worse laboratory findings for albumin, bilirubin, and AFP levels were observed in the PVTT group (3.7 ± 0.5 vs. 3.8 ± 0.5, 1.1 ± 1.6 vs. 1.0 ± 0.8; and 13,176.8 ± 44,232.0 vs. 1185.2 ± 7501.9, respectively; all Ps < 0.05). TACE (42.0%) was the most common first-line treatment in both the PVTT and non-PVTT groups. Of the 86 patients receiving radiotherapy, 77 (50 in the PVTT group and 27 in the non-PVTT group) were primarily treated with a TACE-combined regimen. All of the 12 patients in the PVTT group were initially treated with sorafenib monotherapy, while 19 of the 29 patients in the non-PVTT group received sorafenib therapy combined with concurrent TACE.

**Table 1:** Host and tumor characteristics of the pooled cohort

Variable	Pooled cohort				
	All	Without PVTT	With PVTT	Standardized difference	***P***-value
	(***n*** = 1709)	(***n*** = 1503)	(***n*** = 206)		
Age (years)	57.0 ± 9.3	57.2 ± 9.2	55.5 ± 9.5	0.182	0.013
Male	1392 (81.5)	1211 (80.6)	181 (87.9)	0.201	0.012
Hypertension	451 (26.4)	407 (27.1)	44 (21.4)	0.134	0.081
Etiology of liver disease				0.189	0.072
HBV infection	1437 (84.1)	1254 (83.4)	183 (88.8)		
HCV infection	131 (7.7)	123 (8.2)	8 (3.9)		
Others	141 (8.2)	126 (8.4)	15 (7.3)		
Child-Pugh class					
A	1564 (91.5)	1380 (91.8)	184 (89.3)	0.085	0.228
B	145 (8.5)	123 (8.2)	22 (10.7)		
PT (INR)	1.10 ± 0.12	1.09 ± 0.12	1.10 ± 0.10	0.033	0.115
Albumin (g/dl)	3.8 ± 0.5	3.8 ± 0.5	3.7 ± 0.5	0.192	0.010
Bilirubin (mg/dl)	1.0 ± 0.9	1.0 ± 0.8	1.1 ± 1.6		0.036
Creatinine (mg/dl)	0.9 ± 0.6	0.9 ± 0.5	0.9 ± 1.0	0.072	0.678
Platelet (× 10^3^/uL)	133.1 ± 57.2	132.3 ± 56.2	139.4 ± 63.6	0.120	0.265
AST (IU/L)	51.4 ± 39.6	50.6 ± 38.3	57.0 ± 47.9	0.147	0.204
ALT (IU/L)	42.5 ± 34.6	42.3 ± 33.7	43.7 ± 40.2	0.037	0.755
Ascites	49 (2.9)	31 (2.1)	18 (8.7)	0.299	< 0.001
MELD score	8.2 ± 2.3	8.2 ± 2.3	8.4 ± 2.5	0.086	0.251
Liver cirrhosis	1.709 (100)	1.503 (100)	206 (100)	0.000	0.999
Number of tumors				0.221	0.002
Single	1115 (65.3)	1000 (66.5)	115 (55.8)		
Multiple	594 (34.7)	503 (33.5)	91 (44.2)		
Infiltrative-type tumor	99 (5.8)	23 (1.5)	76 (36.9)	1.004	< 0.001
Tumor size (cm)	3.9 ± 2.8	3.5 ± 2.4	7.2 ± 3.5	1.218	< 0.001
Serum AFP (ng/mL)	2630.6 ± 17,308.0	1185.2 ± 7501.9	13,176.8 ± 44,232.0	0.378	< 0.001
Extrahepatic metastasis	85 (5.0)	42 (2.8)	43 (20.9)	0.583	< 0.001
Primary anti-HCC treatment					
TACE	717 (42.0)	577 (38.4)	140 (68.0)	0.620	< 0.001
Radiotherapy*	86 (5.0)	32 (2.1)	54 (26.2)	0.736	< 0.001
Sorafenib^¥^	41 (2.4)	29 (1.9)	12 (5.8)	0.203	0.002

Values are expressed as mean ± standard deviation, or frequency (percentage)

*PVTT* portal vein tumor thrombosis, *HBV* hepatitis B infection, *HCV* hepatitis C virus, *PT* prothrombin time, *INR* international normalized ratio, *AST* aspartate aminotransferase, *ALT* alanine aminotransferase, *MELD* model for end-stage liver disease, *AFP* alpha-fetoprotein, *HCC* hepatocellular carcinoma, and *TACE* transarterial chemoembolization

^*^Of the 86 patients receiving radiotherapy, 77 were primarily treated with a TACE-combined regimen: 50 in the PVTT group and 27 in the non-PVTT group

^¥^Of the 29 patients receiving sorafenib in the non-PVTT group, 19 were primarily treated with a TACE-combined regimen; while 12 patients with PVTT group only treated with sorafenib regimen

After matching multiple covariates (i.e., age, sex, AST, ALT, PT, platelet, creatinine, hypertension, presence of ascites, HBV infection, and Child-Pugh class) in the pooled cohort, 161 patients with PVTT were matched with 309 controls without PVTT (Supplementary Table 1). Thirteen cases were matched to only one control in order to minimize case exclusion. There were thus 148 PVTT patients with 2 controls, and 13 with only one control. Among the matched pairs, it was evident that the PVTT group had more aggressive tumor characteristics in terms of tumor size (7.1 ± 3.6 vs. 3.6 ± 2.4), serum AFP level (12,514.5 ± 36,419.6 vs. 1025.6 ± 4349.6), multiple HCCs (44.7% vs. 33.7%), and extrahepatic metastasis (18.0% vs. 3.6%; all Ps < 0.05) (Supplementary Table 2).

### Endoscopic findings and preemptive medication in the matched cohort

In the matched-pair analysis, higher percentages of overall esophageal varices (37.9% vs. 26.5%; odds ratio [OR] 1.689, [95% CI, 1.116–2.446], *P* = 0.006) and high-risk varices on initial endoscopic images (23.0% vs. 13.3%; OR, 1.950 [95% CI, 1.262–3.104], *P* = 0.003) were found in the PVTT group than in the non-PVTT group; there were no differences regarding gastric varices or portal hypertensive gastropathy (Table 2). The proportion of patients receiving prophylactic treatment with NSBBs did not differ between the two groups (13.4% vs. 16.8%; *P* = 0.423).

**Table 2:** Comparison of endoscopic findings in the matched cohort

	Without PVTT (reference)	With PVTT	OR	95% CI	***P***-value
Esophageal varices	82 (26.5)	61 (37.9)	1.689	1.116–2.446	0.006
High-risk varices	41 (13.3)	37 (23.0)	1.950	1.262–3.104	0.003
Gastric varices	34 (11.0)	23 (14.3)	1.348	0.794–2.290	0.269
Portal hypertensive gastropathy	24 (7.8)	19 (11.8)	1.589	0.902–2.798	0.109

Values are expressed as frequency (percentage)

*PVTT* portal vein tumor thrombosis, *OR* odds ratio, and *CI* confidence interval

### Bleeding episodes and mortality in the matched cohort

**Fig. 2:**
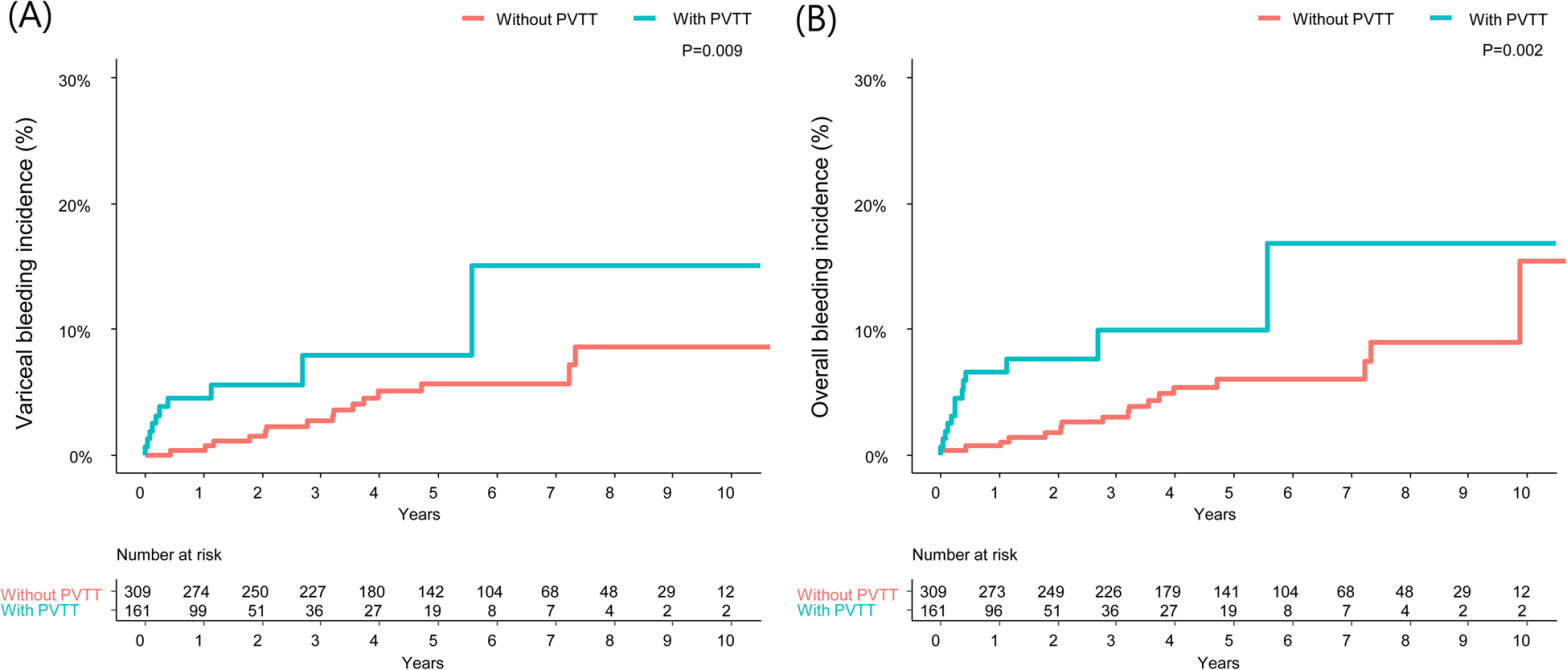
Kaplan-Meier analysis for variceal bleeding and overall bleeding in the matched cohort, according to the presence of PVTT. Significantly higher cumulative incidence of **a** variceal bleeding, as well as **b** overall bleeding was observed in the PVTT group. PVTT, portal vein tumor thrombosis

During a median follow-up of 43.2 months (range, 15.0–71.7 months), 13 (8.1%) and 10 (6.2%) of the PVTT group versus 17 (5.5%) and 15 (4.9%) of the non-PVTT group had overall and variceal bleeding episodes during the observation period, respectively, in the matched cohort (*P* = 0.279 and *P* = 0.534, respectively by Chi-square test). A significantly higher cumulative incidence of variceal bleeding was observed in the PVTT group, compared with the counterpart (4.5% vs. 0.4% at 1 year; 7.9% vs. 2.7% at 3 year; and 7.9% vs. 5.7% at 5 years; HR 2.642, 95% CI [1.270–5.497], *P* = 0.009; Fig. 2a): no bleeding episode was originated from gastric varices alone. An additional multivariate Cox regression analysis also demonstrated that presence of PVTT was an independent risk factor for variceal bleeding in the pooled cohort (HR, 2.525 [95% CI, 1.316–4.843], *P* = 0.005; Supplementary Table 3). A similar pattern emerged in terms of overall hemorrhagic outcome (6.6% vs. 0.7% at 1 year; 10.0% vs. 3.0% at 3 year; and 10.0% vs. 6.0% at 5 year; HR, 2.838 [95% CI, 1.447–5.569], *P* = 0.002; Fig. 2b); five cases with bleeding from gastric or duodenal ulcers (*n* = 3), or angiodysplasia (*n* = 2) were included in the overall bleeding events. As reported in previous studies, [14, 15, 19] median overall survival was significantly shorter in the PVTT group than in the non-PVTT group (1-year survival rates of 65.2% vs. 94.9%; and 3-year survival rates of 25.5% vs. 83.1%, respectively; *P* < 0.001; Supplementary Fig. 1).

### Risk factors associated with high-risk varices and variceal bleeding in the complete set of patients with PVTT

Among the 206 patients with PVTT of whom 38 (18.4%) took NSBBs and 58 (28.2%) had high-risk varices by endoscopy, Vp2, Vp3, and Vp4 disease existed in 76 (36.9%), 48 (23.3%), and 82 (39.8%), respectively. During the study period, acute variceal bleeding and all-cause mortality episodes occurred in 10 (4.9%) and 166 (80.6%), patients, respectively. Almost all bleeding (9/10, 90%) originated from existing high-risk varices, and recurrent variceal bleeding was observed in 2 patients. Variceal bleeding per se did not correlate with long-term overall survival in the population with HCC and PVTT in the multivariate model (Supplementary Table 4). No bleeders died directly of their first episode during hospital stay, during which 8 (80%) received emergent EVL in the early phase within 12 h of evidence of bleeding or admission to the emergency room. In a multivariate analysis of factors related to the presence of high-risk varices at screening in all patients with PVTT, prolonged PT (OR, 1.662 [95% CI, 1.151–2.401], *P* = 0.007), lower platelet count (OR, 0.985 [95% CI, 0.978–0.993], *P* < 0.001), presence of extrahepatic metastasis (OR, 4.240 [95% CI, 1.783–10.084], *P* = 0.001), and higher degree of Vp (Vp4; OR, 3.345 [95% CI, 1.457–7.680], *P* = 0.004) were significant (Table 3). A further multivariate model revealed that presence of high-risk varices (HR, 26.432 [95% CI, 3.230–216.289], *P* = 0.002) and sorafenib use (HR, 5.676 [95% CI, 1.273–25.300], *P* = 0.023) were independent predictors of variceal bleeding (Table 4 and Supplementary Fig. 2).

**Table 3:** Analysis of factors affecting high-risk varices in the entire set of patients with PVTT (*n* = 206)

Variable	None or low-risk varices	High-risk varices	Univariate analysis			Multivariate analysis		
	(***n*** = 148)	(***n*** = 58)	OR	95% CI	***P***-value	OR	95% CI	***P***-value
Age (years)	55.3 ± 9.8	55.9 ± 8.7	1.007	0.976–1.040	0.649			
Male	129 (87.2)	52 (89.7)	1.276	0.483–3.376	0.623			
Hypertension	30 (20.3)	14 (24.1)	1.252	0.608–2.578	0.543			
HBV infection	131 (88.5)	52 (89.7)	1.125	0.420–3.010	0.815			
Child-Pugh class					< 0.001			
A (reference)	139 (93.9)	45 (77.6)	1.0	–	–			
B	9 (6.1)	13 (22.4)	4.462	1.789–11.128	< 0.001			
PT (INR)	1.1 ± 0.1	1.2 ± 0.1	2.170	1.547–3.073	< 0.001	1.662	1.151–2.401	0.007
Creatinine (mg/dl)	0.9 ± 1.0	1.0 ± 0.8	1.027	0.758–1.393	0.863			
Platelet count (×10^3^/uL)	151.0 ± 65.0	109.9 ± 48.9	0.986	0.979–0.990	< 0.001	0.985	0.978–0.993	< 0.001
AST (IU/L)	59.9 ± 52.7	49.6 ± 32.0	0.995	0.987–1.002	0.175			
ALT (IU/L)	45.2 ± 45.6	39.9 ± 21.1	0.996	0.987–1.005	0.410			
Ascites	8 (5.4)	10 (17.2)	3.646	1.360–9.771	0.010			
Number of tumors								
Single (reference)	84 (56.8)	31 (53.4)	1.0	–	–			
Multiple	64 (43.2)	27 (46.6)	1.143	0.621–2.104	0.667			
Tumor size (cm)	7.1 ± 3.5	7.4 ± 3.7	1.020	0.936–1.111	0.651			
Serum AFP (ng/ml)	12,401.0 ± 33,830.0	15,156.6 ± 63,869.8	1.001	0.995–1.008	0.689			
Extrahepatic metastasis	20 (13.5)	23 (39.7)	4.206	2.075–8.523	< 0.001	4.240	1.783–10.084	0.001
Degree of PVTT								
Vp2 (reference)	61 (41.2)	15 (25.8)	1.0	–	–	1.0	–	–
Vp3	41 (27.7)	7 (12.1)	0.694	0.260–1.851	0.466	0.788	0.251–2.469	0.682
Vp4	46 (31.1)	36 (62.1)	3.183	1.559–6.497	0.001	3.345	1.457–7.680	0.004

Values are expressed as mean ± standard deviation, or frequency (percentage)

*PVTT* portal vein tumor thrombosis, *OR* odds ratio, *CI* confidence interval, *HBV* hepatitis B virus, *PT* prothrombin time, *INR* international normalized ratio, *AST* aspartate aminotransferase, *ALT* alanine aminotransferase, and *AFP* alpha-fetoprotein

**Table 4:** Factors predicting variceal bleeding episodes in the entire set of patients with PVTT (*n* = 206)

Variable	Without variceal bleeding	With variceal bleeding	Univariate analysis			Multivariate analysis		
	(***n*** = 196)	(***n*** = 10)	HR	95% CI	***P***-value	HR	95% CI	***P***-value
Age (years)	55.6 ± 9.5	52.9 ± 7.9	0.979	0.916–1.045	0.523			
Male	173 (88.3)	8 (80.0)	2.023	0.428–9.566	0.374			
Hypertension	42 (21.4)	2 (20.0)	0.997	0.210–4.723	0.997			
HBV infection	173 (88.3)	10 (100.0)						
Child-Pugh class								
A (reference)	174 (88.8)	10 (100.0)						
B	22 (11.2)	0 (0.0)						
NSBBs	36 (18.4)	2 (20.0)	1.283	0.270–6.090	0.754			
PT (INR)	1.1 ± 0.1	1.2 ± 0.1	1.956	1.191–3.210	0.008			
Creatinine (mg/dl)	0.9 ± 0.1	0.9 ± 0.2	0.774	0.112–5.355	0.795			
Platelet count (×10^3^/uL)	140.1 ± 64.2	127.2 ± 51.4	0.996	0.985–1.007	0.528			
AST (IU/L)	57.5 ± 48.8	48.3 ± 26.4	0.995	0.976–1.013	0.576			
ALT (IU/L)	43.9 ± 40.8	40.2 ± 29.1	0.997	0.975–1.018	0.758			
Ascites	18 (9.2)	0 (0.0)						
Number of tumors								
Single (reference)	109 (55.6)	6 (60.0)	1.0	–	–			
Multiple	87 (44.4)	4 (40.0)	1.151	0.316–4.194	0.831			
Tumor size (cm)	7.1 ± 3.5	8.4 ± 4.9	1.237	0.258–5.929	0.790			
Serum AFP (ng/ml)	13,429.2 ± 45,167.2	8230.8 ± 18,268.5	1.154	0.976–1.364	0.093			
Extrahepatic metastasis	41 (20.9)	2 (20.0)	0.998	0.976–1.021	0.875			
Degree of PVTT								
Vp2 (reference)	74 (37.7)	2 (20.0)	1.0	–	–			
Vp3	45 (23.0)	3 (30.0)	2.464	0.406–14.924	0.327			
Vp4	77 (39.3)	5 (50.0)	2.965	0.567–15.497	0.198			
Primary anti-HCC treatment								
TACE	133 (67.9)	7 (70.0)	1.174	0.302–4.574	0.817			
Radiotherapy	52 (26.5)	2 (20.0)	0.753	0.160–3.554	0.720			
Sorafenib	9 (4.6)	3 (30.0)	11.615	2.809–48.030	0.001	5.676	1.273–25.300	0.023
High-risk varices	49 (25.0)	9 (90.0)	31.323	3.928–249.782	0.001	26.432	3.230–216.289	0.002

Values are expressed as the mean ± standard deviation, or frequency (percentage)

*PVTT* portal vein tumor thrombosis, *HR* hazards ratio, *CI* confidence interval, *HBV* hepatitis B virus, *NSBB* nonselective beta-blocker, *PT* prothrombin time, *INR* international normalized ratio, *AST* aspartate aminotransferase, *ALT* alanine aminotransferase, *AFP* alpha-fetoprotein, *HCC* hepatocellular carcinoma, and *TACE* transarterial chemoembolization

## Discussion

In this matched study of asymptomatic HCC patients, we found that a quarter of patients with HCC invading the portal vein had high-risk varices in the esophagus in the initial endoscopic work-up, indicated by the presence of high-grade varices or red wale marks. Only about 6% experienced active variceal hemorrhages during the period of HCC treatment, and all but one of these bleeders had high-risk varices. The presence of PVTT was associated with a 2.6-fold higher risk of developing variceal bleeding over time, and this risk was independent of underlying liver function and coagulopathy, as well as the use of NSBBs.

The development of PVTT in patients with HCC is known to be mainly due to direct and contiguous vascular invasion by the tumor [20, 21]. PVTT can cause an increase in portal hypertension, followed by rapid growth of venous collateral vessels, and may contribute to the development or aggravation of gastro-esophageal varices and potential hemorrhagic complications [22]. Clinical investigations have established a positive association between PVTT and high-risk varices and variceal hemorrhage in patients with HCC [8, 9]. This feature was also observed in cirrhotic patients with benign portal vein thrombosis [10]. In terms of primary prophylaxis for silent varices at risk of bleeding, it is currently advised that cirrhotic patients be initiated on NSBBs or considered for EVL [5, 11, 12, 17]. However, no guidelines have been set to deal specifically with the management of varices in patients with HCC, and this is the more unfortunate in that PVTT has been deemed a greater risk [13]. Sorafenib therapy, together with high-risk varices that are inherent risk factors [5], was a strong predictor of bleeding in the ruptured varices of our HCC patients with PVTT, with 30% of variceal bleeders taking the drug. In fact, beneficial roles of sorafenib have been reported in ameliorating portal pressure via it anti-angiogenic and anti-fibrotic effects in animal models [23–25]. However, a recent report revealed an increased risk of all-grade gastrointestinal hemorrhage due to the anti-VEGF effect associated with the use of sorafenib in patients with HCC, especially when the HCC was accompanied by underlying varices, thus affecting the architectural integrity of the endothelial cells of the microvasculature [26]. These findings suggest that although NSBBs may be more cost-effective and easier to administer to prevent first variceal bleeding in most cases with PVTT, [27] endoscopic eradication may be the best option at least in sorafenib-treated patients lacking the hemostatic benefit of portal depression by NSBBs [28, 29].

On the other hand, in our previous study of external beam radiotherapy mainly targeting PVTT induced by HCC we obtained a PVTT response rate of about 40% [30]. Given that resolution of the PVTT after radiotherapy could restore the interrupted portal venous supply of the liver, at least partially rescuing overall liver function, we hypothesized that PVTT shrinkage driven by radiotherapy lowers portal pressure, leading to decreased risk of variceal bleeding. However, radiotherapy did not affect the likelihood of variceal or overall bleeding episodes in our series.

Unexpectedly, we noted that existing high-risk varices and even variceal bleeding had no critical effect on the overall survival of our patients with HCC and PVTT. There have been contradictory findings regarding the effect of variceal bleeding on the long-term survival HCC patients [31–33]. There were no in-hospital deaths following the first active variceal bleeding episode in our subjects with HCC and PVTT, among whom 80% underwent emergent endoscopic therapy within 12 h of onset along with blood volume restitution and hemodynamic stabilization, although one bleeder did die of a serious recurrence of hemorrhage 6 weeks after the initial event. Indeed, early endoscopic confirmation followed by band ligation was able to lower the risk of re-bleeding as well as in-hospital mortality in cirrhotic patients with acute variceal hemorrhage [34, 35].

The retrospective nature of this study has inherent limitations. Specifically, NSBBs were used to prevent the first variceal hemorrhage in only one-third of the patients with high-risk varices. However, medical prophylaxis with NSBBs was not a significant preventive factor for variceal bleeding in our cohort. In fact, approximately 30% of candidates usually have contraindications to NSBBs therapy, or side effects that require cessation of the drug, as shown in prior trials [12].

## Conclusions

In conclusion, HCC patients with PVTT mechanically enhancing portal hypertension are at increased risk of variceal bleeding, although only 15% of individuals with high-risk varices experienced actual episodes. Accordingly, patients with high-risk varices should undergo prophylaxis in essentially the same manner as the general cirrhotic population. In particular, sorafenib users, with the increased risk of bleeding, probably through microvascular disintegration, may prefer to be endoscopically prevented. Optimal prophylactic indications and methods for preventing potential variceal hemorrhage in HCC patients with PVTT should be clarified by prospective studies.

## Supplementary Information

**Additional file 1: Supplementary Table 1.** Demographic and clinical characteristics of the matched cohort. **Supplementary Table 2.** Tumor characteristics of the matched cohort (*n* = 470). **Supplementary Table 3.** Factors predicting variceal bleeding episodes in the pooled cohort (*n* = 1709). **Supplementary Table 4.** Analysis of factors affecting overall survival in the entire set of patients with PVTT (*n* = 206). **Supplementary Figure 1.** Kaplan-Meier analysis for overall survival in matched cohort, according to the presence of PVTT. **Supplementary Figure 2.** Kaplan-Meier analysis for variceal bleeding incidence in the entire patients with PVTT according to the presence of high-risk varices and sorafenib use.

## Data Availability

The datasets generated during the current study are available from the corresponding author on reasonable request.

## Abbreviations

AFP: Alpha-fetoprotein
ALT: Alanine aminotransferase
AST: Aspartate aminotransferase
BCLC: Barcelona Clinic Liver Cancer
CI: Confidence interval
EVL: Endoscopic variceal ligation
HBV: Hepatitis B infection
HCC: Hepatocellular carcinoma
HCV: Hepatitis C virus
HR: Hazard ratio
INR: International normalized ratio
MELD: Model for end-stage liver disease
NSBBs: Non-selective beta-blockers
PT: Prothrombin time
PVTT: Portal vein tumor thrombosis
TACE: Transarterial chemoembolization
