# Supplementary material for: Variceal bleeding is aggravated by portal venous invasion of hepatocellular carcinoma: a matched nested case-control study
Source: BMC Cancer. 2021 Jan 5;21:11. doi: 10.1186/s12885-020-07708-1 (PMC7786454; doi:10.1186/s12885-020-07708-1)
Supplement: Supplementary file 1 — Additional file 1: Supplementary Table 1. Demographic and clinical characteristics of the matched cohort. Supplementary Table 2. Tumor characteristics of the matched cohort (n = 470). Supplementary Table 3. Factors predicting variceal bleeding episodes in the pooled cohort (n = 1709). Supplementary Table 4. Analysis of factors affecting overall survival in the entire set of patients with PVTT (n = 206). Supplementary Figure 1. Kaplan-Meier analysis for overall survival in matched cohort, according to the presence of PVTT. Supplementary Figure 2. Kaplan-Meier analysis for variceal bleeding incidence in the entire patients with PVTT according to the presence of high-risk varices and sorafenib use. [file 12885_2020_7708_MOESM1_ESM.zip › supple_BCAN-D-20-02246.R3.docx]

**Supplementary Materials**

**Table of Contents**

**Supplementary Table 1.** Demographic and clinical characteristics of the matched cohort

**Supplementary Table 2.** Tumor characteristics of the matched cohort (n=470)

**Supplementary Table 3.** Factors predicting variceal bleeding episodes in the pooled cohort (n=1,709)

**Supplementary Table 4.** Analysis of factors affecting overall survival in the entire set of patients with PVTT (n=206)

**Figure of Contents**

**Supplementary Figure 1.** Kaplan-Meier analysis for overall survival in matched cohort, according to the presence of PVTT

**Supplementary Figure 2.** Kaplan-Meier analysis for variceal bleeding incidence in the entire patients with PVTT according to the presence of high-risk varices and sorafenib use

**Supplementary Table 1.** Demographic and clinical characteristics of the matched cohort

| **Variable** | **Matched cohort** | | | | | |  |  |
| --- | --- | --- | --- | --- | --- | --- | --- | --- |
|  | **All** | | **Without PVTT** | | **With PVTT** | **Standardized difference** | | |
|  | **(n = 470)** | | **(n = 309)** | | **(n=161)** |  |  |  |
| Age | 54.7±7.7 | | 54.7±7.5 | | 54.7±8.0 | 0.010 | | |
| Male | 422 (89.8) | | 277 (89.6) | | 145 (90.1) | 0.014 | | |
| Hypertension | | 83 (17.7) | 53 (17.2) | | 30 (18.6) | | 0.039 | |
| HBV infection | 451 (95.9) | | 297 (96.2) | | 154 (95.7) | 0.241 | | |
| Child-Pugh class |  | |  | |  |  | | |
| A | 451 (96.0) | | 297 (96.1) | | 154 (95.7) | 0.023 | | |
| B | 19 (4.0) | | 12 (3.9) | | 7 (4.4) |  | | |
| PT (INR) | 1.1±0.1 | | 1.1±0.1 | | 1.1±0.1 | 0.058 | | |
| Creatinine (mg/dl) | 0.9±0.2 | | 0.9±0.2 | | 0.9±0.2 | 0.048 | | |
| Platelet (×10³/uL) | 138.3±56.6 | | 136.4±52.4 | | 141.8±63.9 | 0.092 | | |
| AST (IU/L) | 50.4±38.2 | | 50.0±37.1 | | 51.3±40.3 | 0.034 | | |
| ALT (IU/L) | 39.7±31.1 | | 40±30.6 | | 39.3±32.2 | 0.022 | | |
| Ascites | 7 (1.5) | | 4 (1.3) | | 3 (1.9) | 0.046 | | |

The values are expressed as the mean ±standard deviation, or frequency (percentage).

PVTT, portal vein tumor thrombosis; HBV, hepatitis B virus; PT, prothrombin time; INR, international normalized ratio; AST, aspartate aminotransferase; ALT, alanine aminotransferase; and MELD, model for end-stage liver disease

**Supplementary Table 2.** Tumor characteristics of the matched cohort (n=470)

| **Variable** | **Matched cohort** | | | | |
| --- | --- | --- | --- | --- | --- |
|  | **All** | **Without PVTT** | **With PVTT** | **Standardized difference** | **P-value** |
|  | **(n = 470)** | **(n = 309)** | **(n=161)** |  |  |
| Number of tumors |  |  |  | 0.228 | 0.020 |
| Single | 294 (62.6) | 205 (66.3) | 89 (55.3) |  |  |
| Multiple | 176 (37.4) | 104 (33.7) | 72 (44.7) |  |  |
| Tumor size (cm) | 4.8±3.3 | 3.6±2.4 | 7.1±3.6 | 1.146 | <0.001 |
| Serum AFP (ng/mL) | 4,961.1±22,242.2 | 1,025.6±4,349.6 | 12,514.5±36,419.6 | 0.443 | <0.001 |
| Extrahepatic metastasis | 40 (8.5) | 11 (3.6) | 29 (18.0) | 0.479 | <0.001 |
| Anti-HCC treatment |  |  |  |  |  |
| TACE | 246 (52.3) | 137 (44.3) | 109 (67.7) | 0.484 | <0.001 |
| Radiotherapy | 49 (10.4) | 4 (1.3) | 45 (28.0) | 0.815 | <0.001 |
| Sorafenib | 9 (1.9) | 0 (0.0) | 9 (5.6) | 0.344 | 0.983 |

The values are expressed as the mean ±standard deviation, or frequency (percentage).

PVTT, portal vein tumor thrombosis; AFP, alpha-fetoprotein; HCC, hepatocellular carcinoma; and TACE, transarterial chemoembolization

**Supplementary Table 3.** Factors predicting variceal bleeding episodes in the pooled cohort (n=1,709)

|  | **Univariate analysis** | | |  | **Multivariate analysis** | | |
| --- | --- | --- | --- | --- | --- | --- | --- |
|  | **HR** | **95% CI** | **P-value** |  | **HR** | **95% CI** | **P-value** |
| Age (years) | 1.001 | 0.977-1.025 | 0.940 |  |  |  |  |
| Male | 0.707 | 0.383-1.304 | 0.267 |  |  |  |  |
| Hypertension | 0.895 | 0.541-1.482 | 0.667 |  |  |  |  |
| HBV infection | 0.446 | 0.271-0.733 | 0.001 |  | 0.458 | 0.277-0.757 | 0.002 |
| Child-Pugh class B | 2.136 | 1.101-4.144 | 0.025 |  |  |  |  |
| PT (INR) | 23.91 | 7.556-75.658 | <0.001 |  | 7.489 | 1.512-37.104 | 0.014 |
| Creatinine (mg/dl) | 1.136 | 0.883-1.461 | 0.321 |  |  |  |  |
| Platelet (×10³/uL) | 0.986 | 0.981-0.991 | <0.001 |  | 0.990 | 0.984-0.995 | <0.001 |
| AST (IU/L) | 0.996 | 0.989-1.003 | 0.267 |  |  |  |  |
| ALT (IU/L) | 0.995 | 0.987-1.003 | 0.244 |  |  |  |  |
| Ascites | 0.813 | 0.113-5.851 | 0.837 |  |  |  |  |
| Presence of PVTT | 2.153 | 1.129-4.105 | 0.020 |  | 2.525 | 1.316-4.843 | 0.005 |

HR, Hazard ratio; CI, confidence interval; HBV, hepatitis B infection; PT, prothrombin time; INR, international normalized ratio; AST, aspartate aminotransferase; ALT, alanine aminotransferase; PVTT, portal vein tumor thrombosis

**Supplementary Table 4.** Analysis of factors affecting overall survival in the entire set of patients with PVTT (n=206)

|  | **Univariate analysis** | | |  | **Multivariate analysis** | | |
| --- | --- | --- | --- | --- | --- | --- | --- |
|  | **HR** | **95% CI** | **P-value** |  | **HR** | **95% CI** | **P-value** |
| Age (years) | 1.008 | 0.992-1.023 | 0.329 |  |  |  |  |
| Male | 1.160 | 0.739-1.818 | 0.519 |  |  |  |  |
| Hypertension | 1.201 | 0.831-1.733 | 0.328 |  |  |  |  |
| HBV infection | 0.472 | 0.299-0.743 | 0.001 |  | 0.567 | 0.350-0.921 | 0.022 |
| Child-Pugh class B | 4.264 | 2.654-6.849 | <0.001 |  | 1.930 | 1.066-3.494 | 0.030 |
| NSBBs | 1.333 | 0.909-1.953 | 0.140 |  |  |  |  |
| PT (INR) | 1.633 | 1.407-1.895 | <0.001 |  | 1.351 | 1.140-1.600 | 0.001 |
| Creatinine (mg/dl) | 1.003 | 0.845-1.190 | 0.969 |  |  |  |  |
| Platelet (×10³/uL) | 1.001 | 0.998-1.003 | 0.552 |  |  |  |  |
| AST (IU/L) | 1.001 | 0.997-1.004 | 0.678 |  |  |  |  |
| ALT (IU/L) | 1.002 | 0.998-1.006 | 0.244 |  |  |  |  |
| Ascites | 2.919 | 1.759-4.843 | <0.001 |  | 2.145 | 1.176-3.915 | 0.013 |
| Multiple tumors | 2.151 | 1.579-2.930 | <0.001 |  | 1.831 | 1.329-2.521 | <0.001 |
| Tumor size (cm) | 1.124 | 1.078-1.171 | <0.001 |  | 1.106 | 1.060-1.154 | <0.001 |
| Serum AFP (ng/ml) | 1.007 | 1.004-1.010 | <0.001 |  |  |  |  |
| Extrahepatic metastasis | 1.970 | 1.377-2.819 | <0.001 |  |  |  |  |
| Degree of PVTT |  |  |  |  |  |  |  |
| Vp2 (reference) | 1.0 | - | - |  |  |  |  |
| Vp3 | 1.568 | 1.036-2.372 | 0.033 |  |  |  |  |
| Vp4 | 2.128 | 1.482-3.052 | <0.001 |  |  |  |  |
| Anti-HCC treatment |  |  |  |  |  |  |  |
| TACE | 1.249 | 0.890-1.752 | 0.198 |  |  |  |  |
| Radiotherapy | 1.289 | 0.924-1.797 | 0.134 |  |  |  |  |
| Sorafenib | 2.132 | 1.152-3.943 | 0.016 |  |  |  |  |
| High-risk varices | 1.595 | 1.147-2.217 | 0.005 |  |  |  |  |
| Variceal bleeding | 2.185 | 1.113-4.288 | 0.023 |  |  |  |  |

PVTT, portal vein tumor thrombosis; HR, hazard ratio; CI, confidence interval; HBV, hepatitis B virus; NSBB, non-selective beta-blocker; PT, prothrombin time; INR, international normalized ratio; AST, aspartate aminotransferase; ALT, alanine aminotransferase; AFP, alpha-fetoprotein; HCC, hepatocellular carcinoma; and TACE, transarterial chemoembolization.

**SUPPLEMENTARY FIGURE LEGENDS**

**Supplementary Figure 1.** Kaplan-Meier analysis for overall survival in matched cohort, according to the presence of PVTT. The analysis included a matched cohort. Patients with PVTT had a significantly shorter life expectation (1-year survival rates of 94.9% vs. 65.2%; and 3-year survival rates of 83.1% vs. 25.5%, respectively; *P*<0.001).

**Supplementary Figure 2.** Kaplan-Meier analysis for variceal bleeding incidence in the entire patients with PVTT according to the presence of high-risk varices and sorafenib use. (A) Presence of high-risk varices at HCC diagnosis, and (B) use of sorafenib were significantly associated with variceal bleeding in HCC patients with PVTT (*P*s<0.001 for both).
