# Supplementary figures and images for: Variceal bleeding is aggravated by portal venous invasion of hepatocellular carcinoma: a matched nested case-control study
Source: BMC Cancer. 2021 Jan 5;21:11. doi: 10.1186/s12885-020-07708-1 (PMC7786454; doi:10.1186/s12885-020-07708-1)

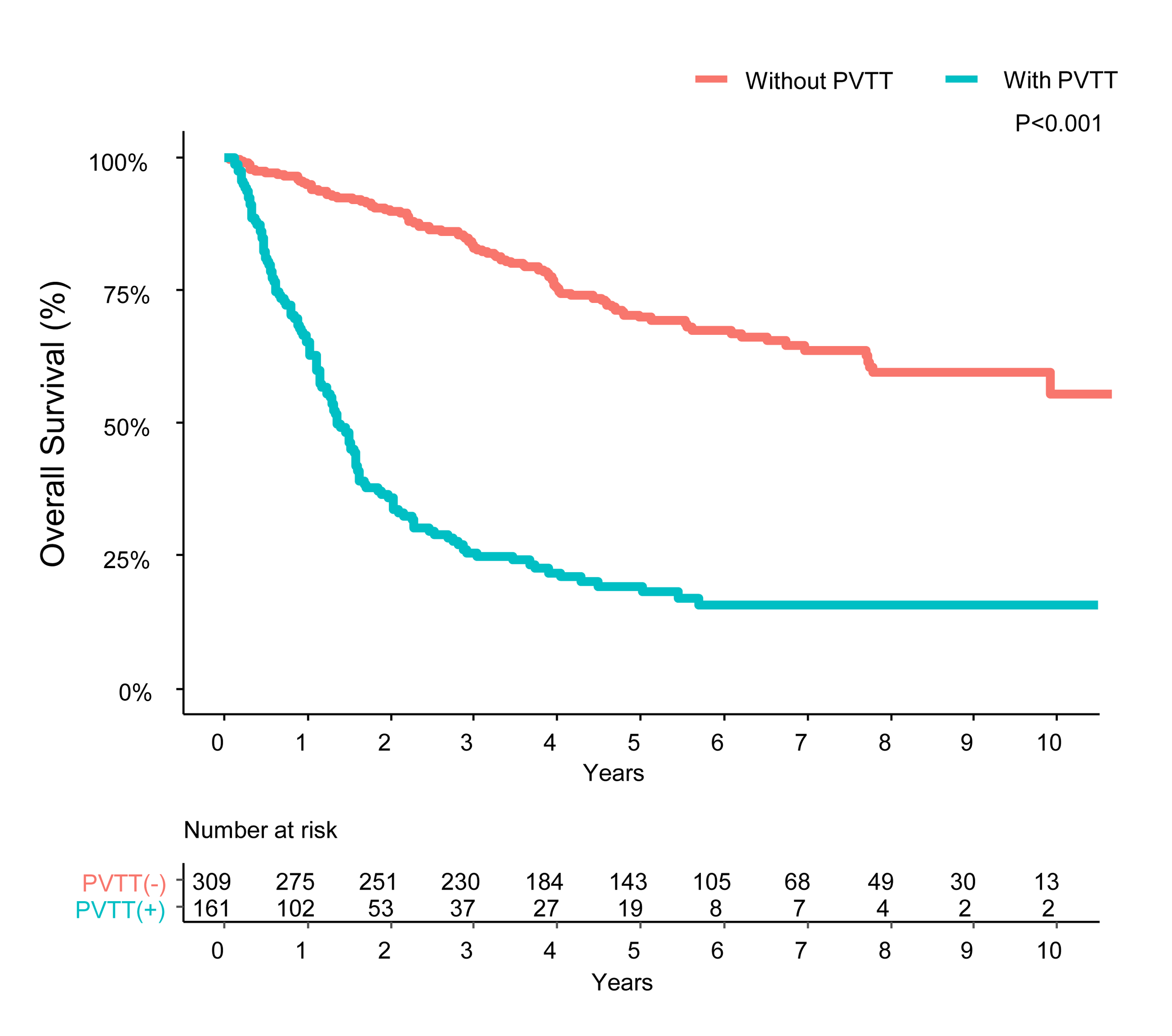

Supplement: Supplementary file 1 — Additional file 1: Supplementary Table 1. Demographic and clinical characteristics of the matched cohort. Supplementary Table 2. Tumor characteristics of the matched cohort (n = 470). Supplementary Table 3. Factors predicting variceal bleeding episodes in the pooled cohort (n = 1709). Supplementary Table 4. Analysis of factors affecting overall survival in the entire set of patients with PVTT (n = 206). Supplementary Figure 1. Kaplan-Meier analysis for overall survival in matched cohort, according to the presence of PVTT. Supplementary Figure 2. Kaplan-Meier analysis for variceal bleeding incidence in the entire patients with PVTT according to the presence of high-risk varices and sorafenib use. [file 12885_2020_7708_MOESM1_ESM.zip › Supple Fig1R3.tif]

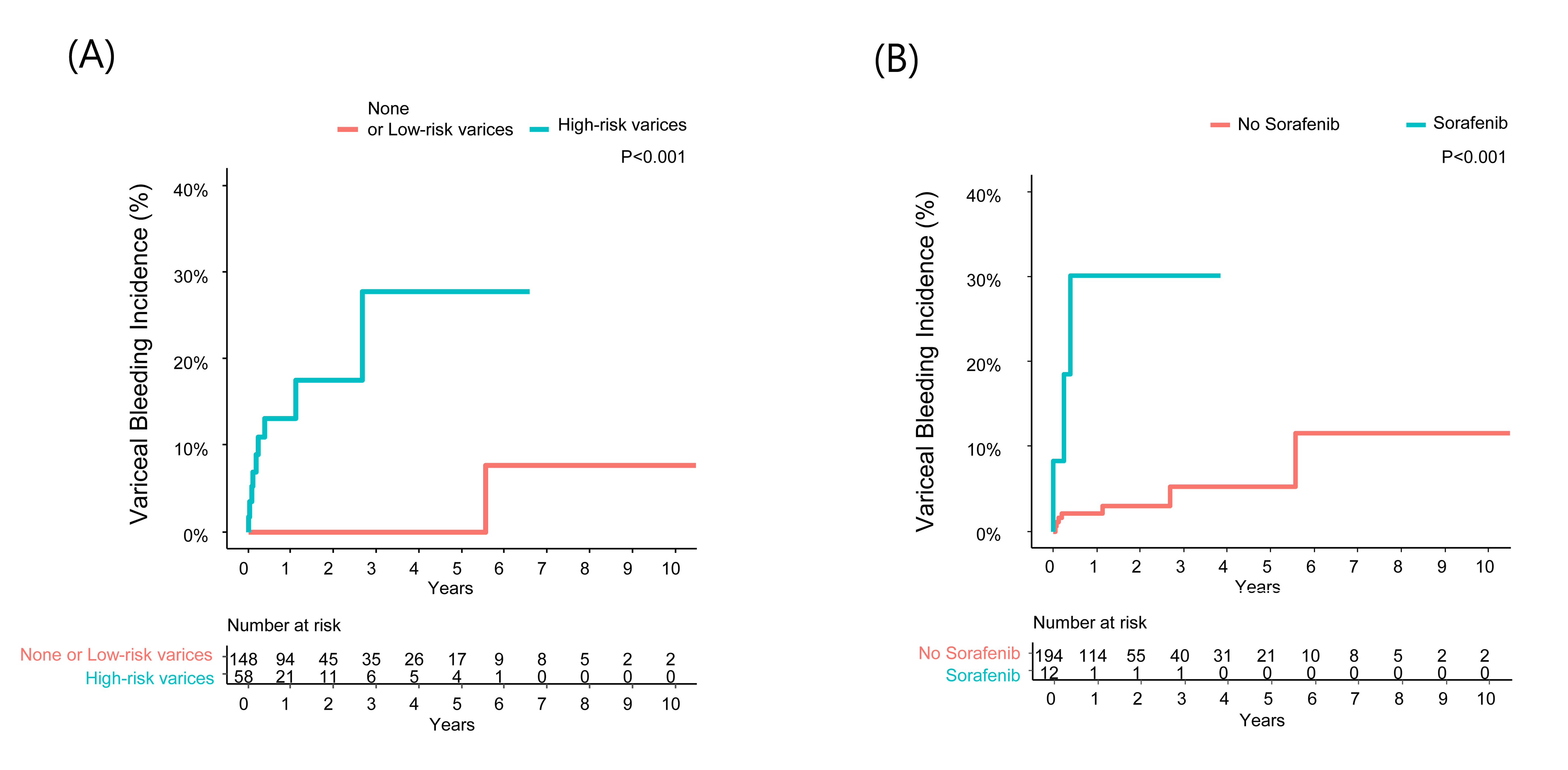

Supplement: Supplementary file 1 — Additional file 1: Supplementary Table 1. Demographic and clinical characteristics of the matched cohort. Supplementary Table 2. Tumor characteristics of the matched cohort (n = 470). Supplementary Table 3. Factors predicting variceal bleeding episodes in the pooled cohort (n = 1709). Supplementary Table 4. Analysis of factors affecting overall survival in the entire set of patients with PVTT (n = 206). Supplementary Figure 1. Kaplan-Meier analysis for overall survival in matched cohort, according to the presence of PVTT. Supplementary Figure 2. Kaplan-Meier analysis for variceal bleeding incidence in the entire patients with PVTT according to the presence of high-risk varices and sorafenib use. [file 12885_2020_7708_MOESM1_ESM.zip › Supple Fig2R3.tif]
